# Supplementary material for: Content-rich biological network constructed by mining PubMed abstracts
Source: BMC Bioinformatics. 2004 Oct 8;5:147. doi: 10.1186/1471-2105-5-147 (PMC528731; doi:10.1186/1471-2105-5-147)
Supplement: Additional File 2 — The original results of the above study (non-essential files are deleted to keep the file size under the limit set by BMC bioinformatics). [file 1471-2105-5-147-S2.bz2 › chilibotAdditionalFile2/dip10/446ID9468473E10452/html/CCR5_CCL8.html]

 


 **CCR5** and **CCL8** 
  
Found 26 abstracts in PubMed, retrieved 10.  
 

 What does Google say? 
 PDF only 
| .edu only 

---

**Interactive relationship** (e.g. stimulation, inhibition, etc)

**Inhibitory relationship**- These data confirm that MCP 2  [ **CCL8** ]  is a ligand for  **CCR5**  on CD4 lymphocytes and can specifically block R5 HIV 1.  Ref: 11930329 J Infect Dis, 2002
- Monocyte chemoattractant protein 2  [ **CCL8** ]  CC chemokine ligand 8  [ **CCL8** ]  inhibits replication of human immunodeficiency virus type 1 via CC chemokine receptor 5  [ **CCR5** ] .  Ref: 11930329 J Infect Dis, 2002
**Stimulatory relationship**- MIP 1alpha, MIP 1beta, RANTES, MCP 2  [ **CCL8** ]  and MCP 4 were able to stimulate increases in intracellular calcium ions via activation of  **CCR5**  whereas MCP 3 was without effect.  Ref: 11861332 Br J Pharmacol, 2002
- The roles of  **CCR5**  in excitotoxic injury in  **CCR5**  deficient mice are compensated by increased CCR2 and CCR3 expression, which share the common MCP 2  [ **CCL8** ]  ligand with  **CCR5** .  Ref: 12807425 J Neurochem, 2003
**Neutral relationship**- Furthermore, MCP 2  [ **CCL8** ]  induces a robust, pertussis toxin sensitive calcium flux in primary lymphocytes, and cross desensitization studies indicate that MCP 2  [ **CCL8** ]  acts via  **CCR5** .  Ref: 11930329 J Infect Dis, 2002
- **CCR5**  is a G protein coupled receptor for RANTES, MIP 1a, MIP 1b and MCP 2  [ **CCL8** ]  that functions as the front line coreceptor for human immunodeficiency virus type 1 infection.  Ref: 12837756 J Biol Chem< MedlineTA>J Biol Chem, 2002
- **CCR5**  is a G protein coupled receptor activated by the chemokines RANTES regulated on activation normal T cell expressed and secreted, macrophage inflammatory protein 1alpha and 1beta, and monocyte chemotactic protein 2  [ **CCL8** ]  and is the main co receptor for the macrophage tropic human immunodeficiency virus strains.  Ref: 11278962 J Biol Chem, 2001
- **CCR5**  is a G protein coupled receptor responding to four natural agonists, the chemokines RANTES regulated on activation normal T cell expressed and secreted, macrophage inflammatory protein MIP 1 alpha, MIP 1 beta, and monocyte chemotactic protein MCP 2  [ **CCL8** ] , and is the main co receptor for the macrophage tropic human immunodeficiency virus strains.  Ref: 12411445 J Biol Chem, 2003

**Non-interactive relationship** (e.g. studied together, co-existance, homology, etc.)

- In order to evaluate if these cells are infectabile by human immunodeficiency virus HIV and could be employed as a model in HIV research we exposed these cell lines to X4 T cell tropic and R5 macrophage tropic and subsequently tried to correlate their infectability with i level of chemokine coreceptor CXCR4 and  **CCR5**  expression, ii coreceptor functionality calcium flux, chemotaxis and phosphorylation of MAPK p42 44 and AKT and iii endogenous expression and secretion of HIV related chemokines which compete with the virus for binding to CXCR4 SDF 1 CXCL12 or  **CCR5**  MIP 1beta CCL4, MIP 1alpha CCL3, RANTES CCL5, MCP 2  [ **CCL8** ]   **CCL8** , MCP 3 CCL7 and MCP 4 CCL13.  Ref: 11737246 Eur J Haematol, 2001
- We investigated the effects of a number of naturally occurring chemokines MIP 1alpha, MIP 1beta, RANTES, MCP 2  [ **CCL8** ] , MCP 3, MCP 4 on different processes linked to the chemokine receptor  **CCR5**  in recombinant CHO cells expressing the receptor at different levels.  Ref: 11861332 Br J Pharmacol, 2002
- A number of inflammatory CC chemokines, including MIP 1 alpha, MIP 1 beta, RANTES, MCP 2  [ **CCL8** ] , and HCC 1 9 74 act as  **CCR5**  agonists, while MCP 3 is a natural antagonist of the receptor.  Ref: 12402506 Receptors Channels, 2002
